# Circulating and vascular NAD^+^ metabolism remodeling in Tgαq*44 mice with progressive heart failure

**DOI:** 10.3389/fmolb.2026.1927197

**Published:** 2026-10-09

**Authors:** Agata Jedrzejewska, Oliwia Krol, Patryk Mucha, Ada Kawecka, Ewa Slominska, Mariola Olkowicz, Ryszard T. Smolenski, Marta Tomczyk

**Affiliations:** 1 Department of Biochemistry, Medical University of Gdansk, Gdansk, Poland; 2 Department of Biopharmaceutics and Pharmacodynamics, Medical University of Gdansk, Gdansk, Poland; 3 Jagiellonian Centre for Experimental Therapeutics (JCET), Jagiellonian University, Krakow, Poland

**Keywords:** CD38, endothelium, heart failure, NAD+ metabolism, Tgαq*44 mice, vascular remodeling

## Abstract

Heart failure (HF) is a complex systemic syndrome in which ongoing myocardial dysfunction and vascular alterations interact to drive disease progression. Given its pivotal role in cellular energetics and redox balance, nicotinamide adenine dinucleotide (NAD^+^) has emerged as a key regulator of cardiovascular homeostasis. We recently demonstrated that myocardial NAD^+^ depletion occurs early in Tgαq*44 mice, a model of chronic HF. Accordingly, this study sought to characterize systemic and vascular alterations in NAD^+^ metabolism throughout the progression of HF by profiling circulating NAD^+^ precursors and metabolites, and assessing vascular NAD^+^ catabolic pattern. In female Tgαq*44 mice aged 4–14 months, whole-blood NAD^+^ and serum nicotinamide (NAM) levels progressively declined with increasing HF severity and were strongly associated with deteriorating systolic function. Reduced circulating NAD^+^ availability coincided with enhanced aortic NAD^+^ turnover and increased aortic CD38 abundance in end-stage HF. In addition, we identified a low-molecular-weight VCAM-1-immunoreactive band in the aorta, consistent with enhanced vascular activation in Tgαq*44 mice. Advanced HF was also associated with perturbations in arginine-citrulline metabolism, characterized by elevated levels of methylated arginine derivatives that may compromise nitric oxide (NO) bioavailability. This was paralleled by progressive accumulation of 4-pyridone-3-carboxamide ribonucleoside (4PYR), an NAD^+^-derived endothelial toxin, whose levels correlated with all three methylarginines, suggesting convergent pathways of endothelial dysfunction. Collectively, these findings suggest that enhanced vascular NAD^+^ catabolism, associated with increased CD38 abundance, constitutes a previously unrecognized metabolic feature of HF progression that may contribute to systemic NAD^+^ dysregulation and endothelial dysfunction.

## Graphical Abstract

**Figure:**
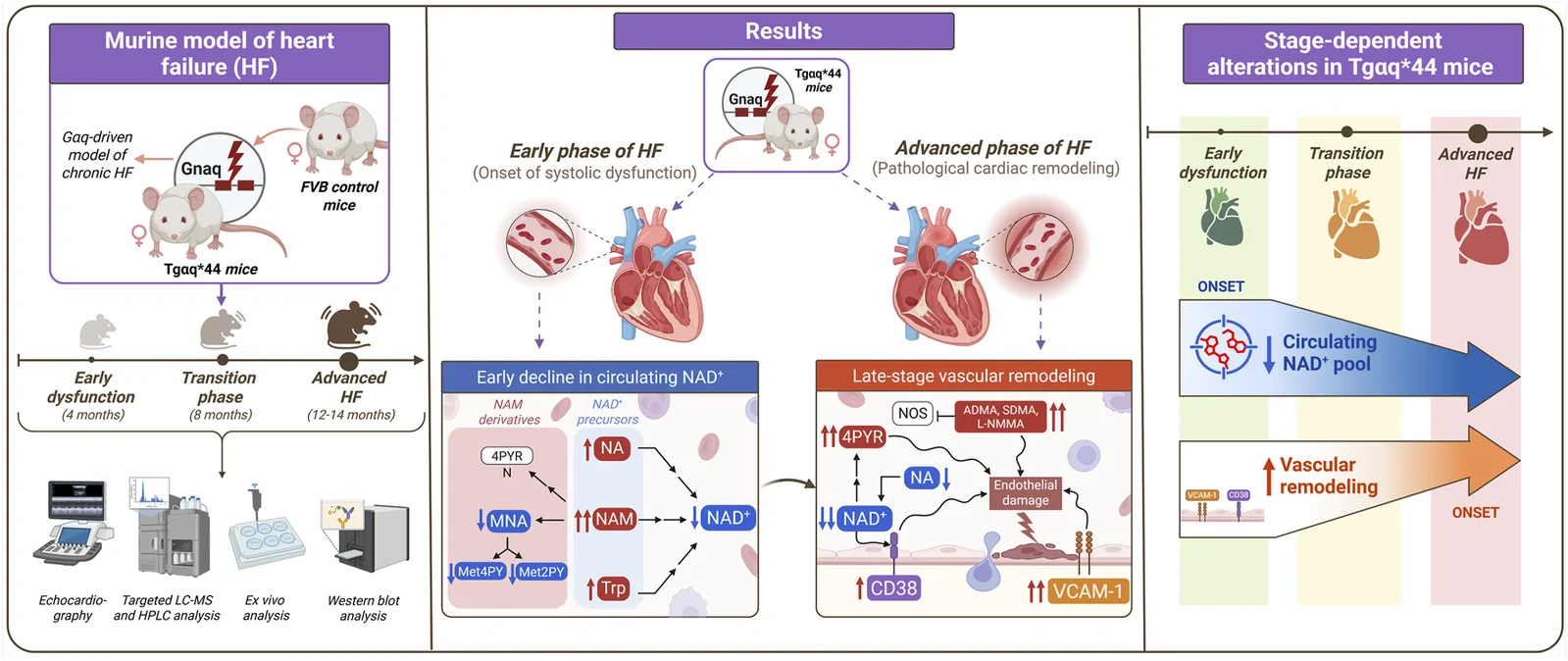
Created in https://BioRender.com.

## 1 Introduction

Heart failure (HF) is a complex systemic syndrome in which inadequate cardiac output leads to tissue hypoxia and metabolic insufficiency. In this context, vascular function and, more broadly, communication between the failing heart and peripheral organs are crucial determinants of disease progression (Yang et al., 2015). However, a growing body of evidence indicates that HF is accompanied by maladaptive vascular abnormalities triggered by either neurohormonal activation or systemic inflammation (Drera et al., 2024). Progressive endothelial dysfunction, which is characterized by reduced nitric oxide (NO) bioavailability and increased oxidative stress, further impairs vascular tone and permeability (Premer et al., 2019). This, in turn, may disrupt the exchange of metabolic substrates and signaling molecules, thereby compromising cardiac performance and contributing to disease worsening (Cornuault et al., 2022). Accordingly, increasing attention has been directed toward mechanisms linking cardiac dysfunction with systemic vascular remodeling and endothelial metabolism (Pietschner et al., 2023; Carulli et al., 2025).

Nicotinamide adenine dinucleotide (NAD^+^) is a central and multifunctional molecule for myocardial homeostasis (Matasic et al., 2018). As an electron carrier, it participates in cellular energy production, whereas as a substrate for sirtuins and poly (ADP-ribose) polymerases, it contributes to the regulation of mitochondrial function, redox balance, and DNA repair (Fang et al., 2023). Collectively, maintenance of the NAD^+^ pool is important for functional and cellular integrity, particularly in a tissue with such high metabolic demands as the heart (Koay et al., 2024). However, NAD^+^ metabolism is also highly relevant to the vascular wall (Jablonska et al., 2021). Although endothelial cells rely predominantly on glycolysis, NAD^+^ availability supports mitochondrial signaling, calcium homeostasis, and NAD^+^-dependent pathways involved in the regulation of NO production. (Wilson et al., 2019). Furthermore, endothelial sirtuins utilize NAD^+^ to promote angiogenesis and enhance NO production, thereby improving blood flow to peripheral organs (Das et al., 2018; Mattagajasingh et al., 2007). Hence, sustained NAD^+^ availability is essential for endothelial proliferation, mitochondrial signaling, and protective responses, whilst NAD^+^ deficiency may contribute to reduced NO synthesis and endothelial dysfunction (Coutelle et al., 2014; Lapel et al., 2017; Xiao et al., 2021; Qiu et al., 2023).

Recently, we demonstrated a marked depletion of the myocardial NAD^+^ pool across the progression of chronic HF in Tgαq*44 mice (Olkowicz et al., 2026). Concomitantly, this model undergoes profound metabolic remodeling, characterized by impaired oxidation of auxiliary fuels and disrupted intermediary metabolism, mirroring metabolic alterations observed in patients with HF. However, it remains unknown whether myocardial NAD^+^ depletion in the Tgαq*44 model is accompanied by changes in circulating NAD^+^ or activation of vascular NAD^+^ catabolism. As discussed above, NAD^+^ imbalance may affect not only cardiomyocyte metabolism but also endothelial function during HF progression. In the present study, we therefore investigated circulating and aortic NAD^+^ metabolism in Tgαq*44 mice across disease progression. We profiled whole-blood nucleotides, serum NAD^+^ precursors and metabolites, as well as amino acid (AA) markers of arginine/NO metabolism. Additionally, we correlated metabolic data with parameters of cardiac function to assess their potential association with HF progression. Then, we examined extracellular NAD^+^/NMN degradation at the aortic surface together with aortic CD38 abundance and vascular adhesion markers.

## 2 Materials and methods

### 2.1 Animals and study design

All experiments were conducted on female Tgαq*44 mice and age-matched control mice (FVB) with the approval of the Local Ethics Committee (Bydgoszcz, Poland; permit no. 43/2025). Tgαq*44 mice develop a slowly progressive, mixed systolic-diastolic cardiac dysfunction, with the 12–14-month period representing the late stage of chronic HF, according to the established characterization of this model (Berkowicz et al., 2023; Tyrankiewicz et al., 2021a). The choice of sex was justified by the characteristics of the Tgαq*44 model, as well as by our previous observations and published reports (Tyrankiewicz et al., 2021b; Widacha et al., 2025). Male Tgαq*44 mice exhibit pronounced aggressive behavior, which potentially may interfere with functional assessments and increase the variability of the results. Accordingly, female animals have been consistently used in longitudinal studies of this model. Although the use of females was not intended to reproduce a female-specific HF phenotype, this choice is clinically relevant in the context of recognized sex-related heterogeneity of HF. Animals were obtained from the Institute of Experimental and Clinical Medicine of the Polish Academy of Sciences (Warsaw) and held in accordance with the Regulation of the Ministry of Agriculture and Rural Development (issued on 22 April 2022). Mice were housed under a 12-h light/dark cycle in individually ventilated cages (IVC) with an area of 432 cm^2^, at 22.5 °C ± 0.5 °C, 55% ± 5% humidity, and unlimited access to water, standard chow diet, and toys (cardboard tunnels and houses). Each IVC provided a minimum of 50 air changes/hour, with filtration and constant airflow of approximately 0.1 m/s. The animal facility maintained at least 15 air changes per hour. At the target age, body mass was measured. Next, mice were anesthetized via intraperitoneal injection of a ketamine/xylazine mixture (50 mg/kg and 5 mg/kg, respectively; Biowet Puławy, Poland) and underwent echocardiography. In the absence of a tactile response, the skin was incised, and blood (35–50 μL) as well as aorta samples were collected and immediately snap-frozen in liquid nitrogen or used in *ex vivo* analysis (see Section 2.5). The remaining blood was centrifuged at 1,200 × g for 15 min at room temperature to obtain serum. Whole blood, serum, and aorta were stored at −80 °C for subsequent analyses.

### 2.2 Echocardiographic evaluation of left ventricular function and remodelling

Following ketamine/xylazine anesthesia and chest hair removal, mice were positioned on a heating platform to sustain a body temperature of 37 °C. The 21-MHz probe was positioned on the anterior chest wall and the heart was visualized in 2D mode. Each examination was completed within 5 min using a high-resolution ultrasound imaging system for small animals (Vinno 6, VINNO Technology, Suzhou, China). Acquired images were analyzed to determine the hemodynamic parameters of the left ventricle, as previously described (Tomczyk et al., 2022). Left ventricular mass index (LVMI) was calculated as left ventricular mass indexed to body weight. Relative wall thickness (RWT) was calculated as the ratio of 2 x left ventricular posterior wall thickness in diastole (LVPWd) divided by left ventricular internal diameter in diastole (LVIDd). In turn, diastolic function could not be reliably assessed because transmitral-flow and tissue-Doppler recordings of sufficient quality for calculating the E/A and E/e′ ratios were unavailable across all age groups of Tgαq*44 mice. As previously reported, assessment of the E/A ratio in older Tgαq*44 mice was limited by frequent fusion of the E and A waves or the absence of a detectable A wave (Berkowicz et al., 2023; Tyrankiewicz et al., 2021a).

### 2.3 Measurement of nucleotides concentration in whole blood and aorta

Frozen blood samples were extracted with 1.3 M HClO_4_ at a 1:1 (v/v) ratio and centrifuged at 14,000 rpm for 10 min at 4 °C. The resulting supernatants were neutralized with 3 M K_3_PO_4_ to a pH of 5.5–6.5. Frozen aortic fragments were freeze-dried for 24 h and subsequently extracted with 100 μL of 0.4 M HClO_4_ using a glass homogenizer. The homogenates were centrifuged at 14,000 rpm for 10 min at 4 °C, and the resulting supernatants were neutralized with 2 M KOH to a pH of 5.5–6.5. Following a second centrifugation (14,000 rpm, 10 min, 4 °C), the final supernatants from both aortic and blood samples were collected for HPLC analysis (Nexera LC40, Shimadzu, Japan) according to a previously published protocol (Smolenski et al., 1990). NADH was quantified indirectly as adenosine 5′-diphosphoribose (ADPR), the primary product of acidic cleavage of NADH during tissue extraction (Karaś et al., 2024). The adenylate energy charge (AEC) was calculated as the ratio of the sum of ATP and one-half of ADP to the total adenylate pool (TAN; ATP + ADP + AMP), reflecting energy status in the aorta (Atkinson, 1968). Nucleotide concentrations are expressed as μmol/g protein (determined using the BCA assay kit; Novagen kit, Millipore, MA, United States) for aortic tissue and as μM for whole blood.

### 2.4 Quantification of serum AA and nicotinamide (NAM) derivatives

Serum AA and NAM derivative concentrations were determined according to previously published protocols, with minor modifications (Olkowicz et al., 2017; Slominska et al., 2006). Briefly, 25 μL of serum was mixed with 5 μL of the internal standard (2-chloroadenosine) and extracted with methanol to achieve a final concentration of 80% (v/v). Samples were centrifuged twice at 14,000 rpm for 10 min at 4 °C, and the resulting supernatant was evaporated. The residue was reconstituted in 0.1% formic acid at a volume equal to the initial serum volume, centrifuged (14,000 rpm, 10 min, 4 °C), and analyzed by LC-MS (TSQ Vantage EMR, ThermoFisher, United States).

### 2.5 Analysis of NAD^+^ and nicotinamide mononucleotide (NMN) catabolism on the aortic surface

According to a previously published protocol, isolated segments of the thoracic and abdominal aorta were meticulously dissected free of perivascular adipose and surrounding connective tissue in cold HBSS (21-023-CV, Corning, United States) and longitudinally opened to expose the endothelial surface (Jablonska et al., 2021). Cleaned aortic fragments were preincubated in 1 mL HBSS at 37 °C for 10 min in a 24-well plate. Samples were collected before and after 60, 90, and 120 min of incubation with NAD^+^ or NMN (final concentration 50 μM in HBSS) and analyzed for metabolic changes by HPLC. Data are presented as the mean of thoracic and abdominal aorta samples across three time points, normalized to wet tissue weight and incubation time (nmol/min/g of wet tissue).

### 2.6 Assessment of aortic CD38 and adhesion molecule abundance

Aorta-derived protein levels of CD38, intercellular adhesion molecule 1 (ICAM-1), and vascular cell adhesion molecule 1 (VCAM-1) were quantified using the Jess Automated Western blot (ProteinSimple, Bio-Techne, MN, United States). Briefly, aortas were homogenized in 100 μL of RIPA lysis buffer (Sigma-Aldrich, St. Louis, MO, United States) supplemented with a protease inhibitor cocktail (Sigma-Aldrich, St. Louis, MO, United States), using a glass homogenizer. Lysates were incubated on ice for 30 min and clarified by centrifugation at 14,000 rpm for 10 min at 4 °C. Total protein concentration was determined using a BCA protein assay kit (Novagen kit, Millipore, MA, United States) and samples were diluted to equalize protein concentrations according to the manufacturer’s instructions. Primary antibodies against mouse CD38 (Novus Biologicals, Centennial, CO, United States, catalog no. NBP2-25250), mouse β-actin (R&D Systems, Minneapolis, MN, United States, catalog no. MA B8929), mouse glyceraldehyde-3-phosphate dehydrogenase (GAPDH; Novus Biologicals, Centennial, CO, United States, catalog no. NB600-502), rabbit ICAM-1 (Proteintech, Rosemont, IL, United States, catalog no. 10020-1-AP), and rabbit VCAM-1 (Invitrogen, Thermo Fisher Scientific, Waltham, MA, United States, catalog no. MA5-31965) were used at a 1:100 dilution. HRP-conjugated secondary antibodies (anti-mouse IgG-HRP and anti-rabbit IgG-HRP; ProteinSimple, Bio-Techne, Minneapolis, MN, United States) were applied as recommended by the manufacturer. Signal intensities were quantified in Compass software (ProteinSimple). Target proteins were normalized to β-actin in samples from 14-month-old mice and to GAPDH in samples from 4-month-old mice. For VCAM-1, the low- and high-molecular-weight immunoreactive bands were quantified separately according to their apparent molecular weights.

### 2.7 Statistical analyses

Statistical analyses were performed in GraphPad Prism 10.6 (GraphPad Software, San Diego, CA, United States) and RStudio (version 4.4.3; R Foundation for Statistical Computing). Comparisons between transgenic and age-matched wild-type mice (Tgαq*44 vs. FVB) were carried out using an unpaired Welch’s two-sample t-test or the Mann-Whitney test when normality was not met. Normality was assessed using the Shapiro-Wilk test. For the correlation analysis, Pearson’s or Spearman’s test was used depending on data normality. A p-value <0.05 was considered statistically significant. Principal component analysis (PCA) of serum AA concentrations was conducted using centred and scaled variables (z-scores). PC1 vs. PC2 score plots were generated with 95% confidence ellipses. Differences in AA profiles between groups were assessed using permutational multivariate analysis of variance (PERMANOVA). The heatmap was created using log_2_ fold changes of mean metabolite values between the Tgαq*44 and FVB groups. For pathway analysis, AA concentrations for each animal were first z-score transformed, and module scores were then calculated as the sum of metabolites assigned to each module: branched-chain AAs (BCAAs; valine, leucine and isoleucine), aromatic AAs (phenylalanine, tyrosine and tryptophan), glycine/serine/threonine metabolism (glycine, serine and threonine), and nitrogen metabolism (glutamine, glutamate, asparagine, aspartate, and alanine). The NO-dysregulation score was calculated as the sum of z-scores of ADMA, SDMA and L-NMMA relative to arginine availability. Modules were defined based on KEGG pathway annotations and prior literature. Module effects were expressed as the mean difference in module scores between Tgαq*44 and FVB mice, with 95% confidence intervals.

## 3 Results

### 3.1 Progression to HF was characterized by systemic NAD^+^ deficiency and reduced availability of its precursors

Echocardiographic assessment was performed in Tgαq*44 mice during the early (4 months), transition (8 months), and advanced (12–14 months) phases to document progressive deterioration of cardiac performance (Figure 1A). As early as 4 months of age, Tgαq*44 mice exhibited impaired systolic function, defined as left ventricular ejection fraction (EF) and fractional shortening (FS). This was accompanied by pathological remodeling including left ventricular (LV) dilation and altered wall geometry, as reflected by increased LVMI, RWT, and LVPWd. The transition phase was characterized by a further decline in EF and FS, together with the first significant changes in LV cavity dimension and diastolic interventricular septal thickness (IVSd). With progression to advanced HF, systolic performance deteriorated markedly, and substantial pathological LV remodelling became evident. By 14 months of age, pathological remodeling reached its maximal extent, along with significant changes in LVPWd of Tgαq*44 mice. Systolic interventricular septal thickness (IVSs) and systolic posterior wall thickness (LVPWs) did not differ during HF progression in Tgαq*44 mice compared to age-matched FVB control mice. Body weight and LV mass of Tgαq*44 and FVB mice at 4, 8, 12, and 14 months of age are presented in Supplementary Table S1 (Supplementary Material).

**FIGURE 1:**
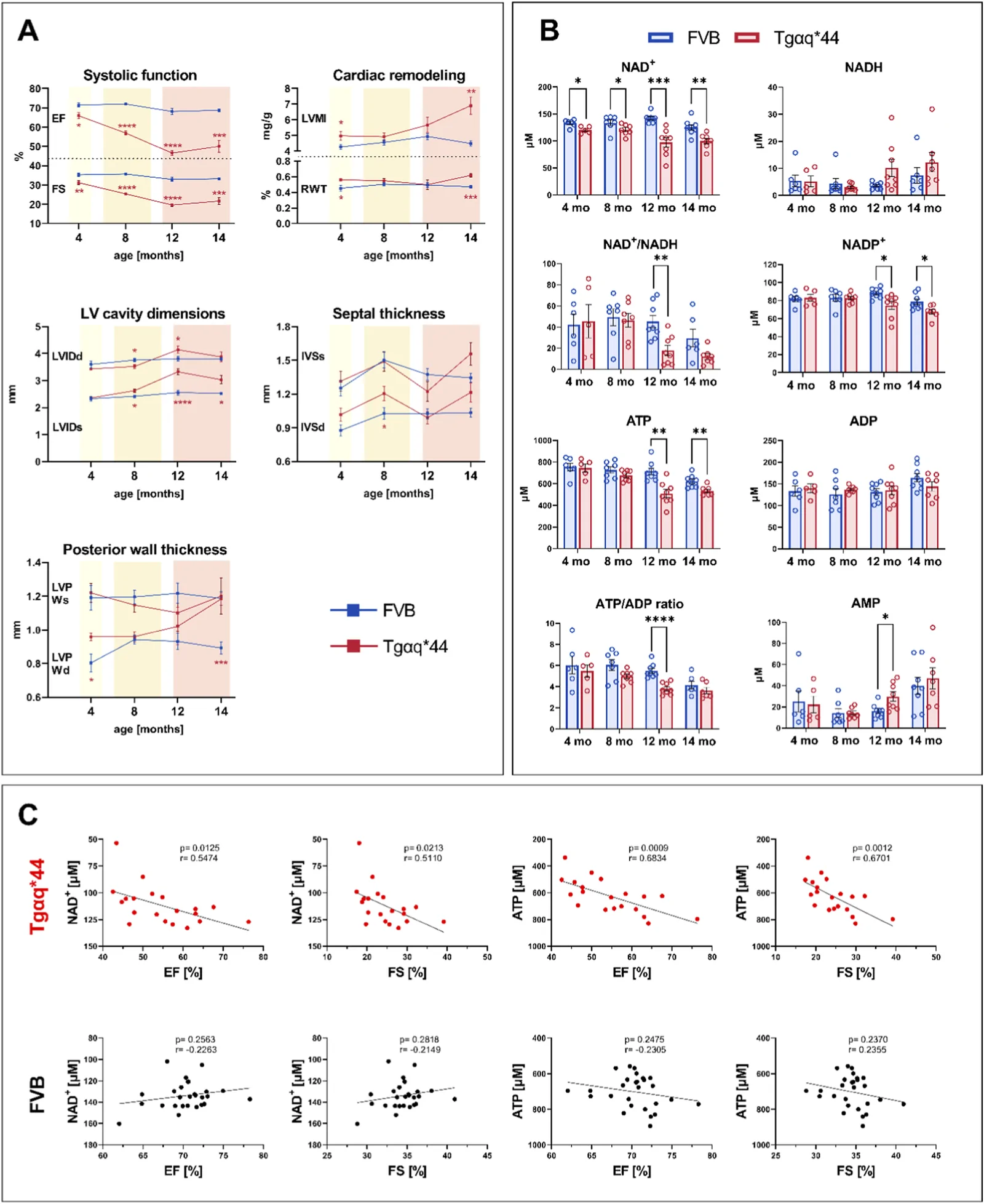
Circulating NAD^+^ and ATP levels decline with the progression of heart failure and are associated with worsening systolic function in Tgαq*44 mice. **(A)** Cardiac function: systolic function (ejection fraction, EF; fractional shortening, FS), cardiac remodeling (left ventricular mass index, LVMI; relative wall thickness, RWT), left ventricular (LV) cavity dimensions (LV internal diameter in diastole and systole, LVIDd/s), septal thickness (interventricular septal thickness in diastole and systole, IVSd/s), and posterior wall thickness (LV posterior wall thickness in diastole and systole, LVPWd/s). **(B)** Whole-blood concentrations of NAD^+^, NADH, NADP^+^, ATP, ADP, and AMP in Tgαq*44 and FVB mice. **(C)** Correlations of whole-blood NAD^+^ and ATP concentrations and systolic function parameters in Tgαq*44 and FVB mice. For panels A and B, data are presented as mean ± SEM, with n = 5–8 mice per age group. *p < 0.05, **p < 0.01, ***p < 0.001, and ****p < 0.0001 vs. FVB by unpaired Welch’s two-sample t-test or Mann-Whitney test. For correlation analyses, red indicates statistically significant correlations. These were determined by Pearson’s or Spearman’s correlation analysis, with n = 20–28 mice per genotype.

Targeted nucleotide analysis revealed a significant decline in peripheral whole-blood NAD^+^ levels in Tgαq*44 mice compared with FVB controls (Figure 1B). Reduced whole-blood NAD^+^ concentrations were observed at the early stage of cardiac dysfunction and remained evident at the advanced stage of HF. By contrast, NADP^+^ concentrations were reduced only at the advanced stage of HF, accompanied by an altered whole-blood NAD^+^/NADH ratio at 12 months of age. Adenine nucleotide levels were also perturbed during established HF, as reflected by lower ATP levels, a reduced ATP/ADP ratio, and increased AMP levels in Tgαq*44 blood.

To strengthen the link between metabolic imbalance and disease progression, we correlated the measured nucleotides with parameters of cardiac efficiency. Significant positive correlations of blood NAD^+^ and ATP levels with systolic function parameters (EF and FS) were observed only in the Tgαq*44 group and were absent in control mice (Figure 1C). Other related nucleotides also correlated with echocardiographic features in the Tgαq*44 group, as shown in the Supplementary Material (Supplementary Table S2). Specifically, NADH and AMP showed opposite trends with EF and FS, whereas the NAD^+^/NADH and ATP/ADP ratios were positively correlated with these parameters. Beyond systolic function, a negative correlation was found between NADH and LVPWs, whilst NAD^+^/NADH and ATP were inversely correlated with LVIDs in Tgαq*44 mice.

To assess changes in NAD^+^ availability, we quantified serum concentrations of NAD^+^ precursors, including nicotinic acid (NA) and NAM (Figure 2A). At the early stage of cardiac dysfunction (4 months), NA and NAM levels were higher in Tgαq*44 mice than in FVB controls. These changes were accompanied by reduced levels of methylated nicotinamide metabolites, including methylnicotinamide (MNA), N-methyl-2-pyridone-5-carboxamide (Met2PY) and N-methyl-4-pyridone-5-carboxamide (Met4PY), but no differences in these derivatives were observed at later disease stages. In turn, NA levels were significantly reduced at 12 months, whereas NAM showed a trend toward lower levels at 12 and 14 months in comparison between Tgαq*44 and control mice. Conversely, the concentration of 4-pyridone-3-carboxamide ribonucleoside (4PYR), a downstream metabolite of NAM, increased progressively during disease progression and was significantly elevated in Tgαq*44 mice at 12 and 14 months.

**FIGURE 2:**
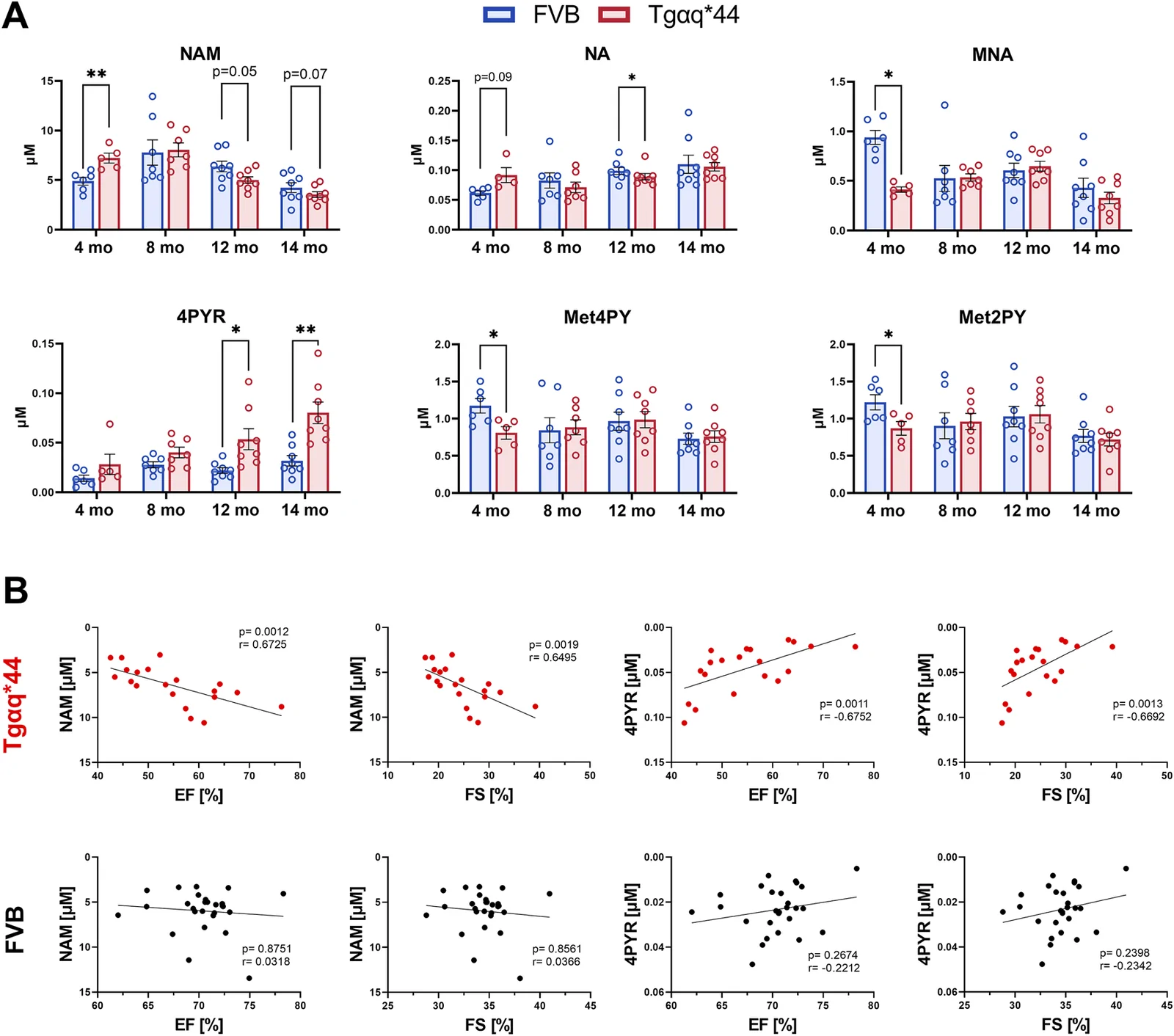
Altered serum NAD^+^ precursor and nicotinamide derivative profiles are associated with cardiac dysfunction in Tgαq*44 mice. **(A)** Serum levels of nicotinic acid (NA), nicotinamide (NAM), N-methylnicotinamide (MNA), N-methyl-2-pyridone-5-carboxamide (Met2PY), N-methyl-4-pyridone-3-carboxamide (Met4PY) and 4-pyridone-3-carboxamide-1-β-D-ribonucleoside (4PYR). **(B)** Correlations between serum NAM and 4PYR concentrations and systolic function parameters (EF, FS) in Tgαq*44 and FVB mice. Data are presented as mean ± SEM; n = 5–8, *p < 0.05, **p < 0.01 vs. FVB by unpaired Welch’s t-test or Mann-Whitney test **(A)**. Red indicates statistically significant correlations determined by Pearson’s or Spearman’s correlation analysis, with n = 20–28 mice per genotype **(B)**.

Correlation analysis further demonstrated that changes in NAD^+^ availability were associated with cardiac deterioration in Tgαq*44 mice (Figure 2B). In particular, lower NAM levels correlated with reduced EF and FS in Tgαq*44 mice, whereas no associations were observed in FVB controls. Unlike NAM, higher 4PYR concentrations were accompanied by lower EF and FS in Tgαq*44 mice only. Additionally, lower NAM levels were associated with more pronounced pathological structural remodeling, as reflected by negative correlations between NAM and both LVIDs and LVMI (Supplementary Table S3, Supplementary Material). Together, these findings are consistent with stage-associated remodeling of NAM metabolism, characterized by lower circulating precursor availability and accumulation of the potentially endothelial-toxic metabolite 4PYR at the advanced disease stage. Within the Tgαq*44 group, this metabolic pattern was associated with impaired systolic function and cardiac structural remodeling.

### 3.2 NAD^+^ turnover was enhanced at the aortic surface in Tgαq*44 mice

To assess extracellular NAD^**+**^ metabolism in Tgαq*44 mice, we measured *ex vivo* NAD^+^ consumption and turnover of its precursor, NMN, using aortic fragments incubated in supplemented medium (Figure 3A). Analysis of NAD^+^ degradation revealed significantly higher hydrolytic activity in the aortic fragments from Tgαq*44 mice at 12 and 14 months of age compared with controls (Figures 3B,C). This may reflect the activity of ecto-nucleotide pyrophosphatase/phosphodiesterase 1 (eNPP1) and/or CD38, which hydrolyse NAD^+^ to NMN and NAM, respectively. However, extracellular NMN production was not different in the Tgαq*44 model compared to the control at any age (Figure 3D). Similarly, AMP (a co-product of eNPP1) was not elevated (Figure 3G), suggesting that eNPP1 is unlikely to be a major contributor to NAD^+^ utilization in this model. In turn, NAM and nicotinamide riboside (NR) were extensively released into the medium with Tgαq*44 aortas collected from 12–14 month-old animals compared with controls (Figures 3E,F). These findings are consistent with a potential contribution of CD38 to NAD^+^ degradation in transgenic endothelium, with subsequent formation of NR potentially mediated by 5′-nucleotidase (CD73) and/or alkaline phosphatase (ALP). Nevertheless, adenosine levels remained unchanged, suggesting that NMN hydrolysis predominated over AMP conversion under these conditions (Figure 3H).

**FIGURE 3:**
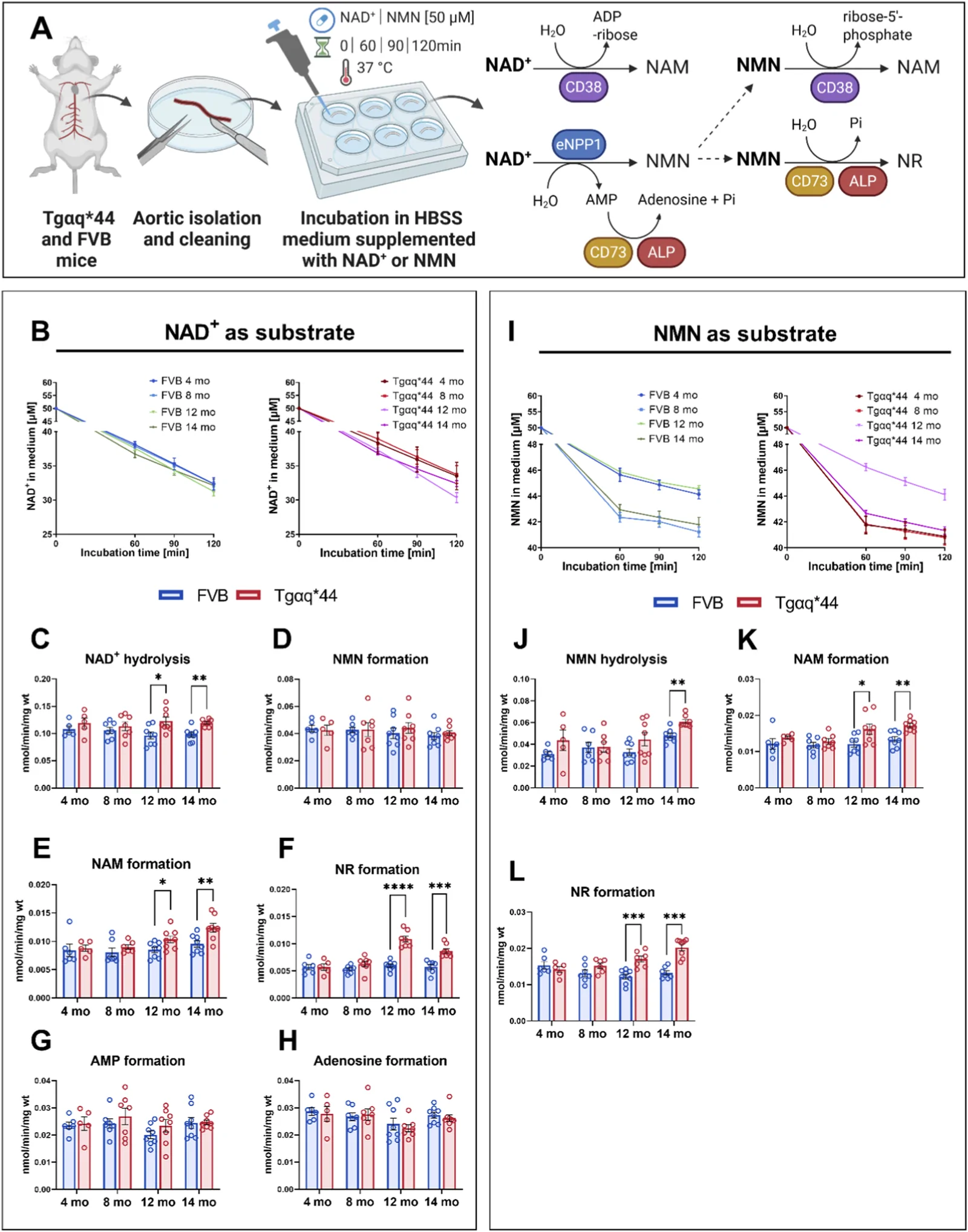
Increased NAD^+^ and NMN catabolism in isolated aortic segments in Tgαq*44 mice with advanced heart failure. **(A)** Experimental design. Concentration-time profiles of NAD^+^ **(B)** and NMN **(I)** after 0, 60, 90, and 120 min of incubation with aortic fragments from FVB and Tgαq*44 mice at 4, 8, 12, and 14 months of age. Rates of NAD^+^ hydrolysis **(C)**, NMN formation **(D)**, NAM formation **(E)**, NR formation **(F)**, AMP formation **(G)**, and adenosine formation **(H)** following NAD^+^ addition. Rates of NMN hydrolysis **(J)**, NAM formation **(K)**, and NR formation **(L)** following NMN addition. Data are presented as mean ± SEM; n = 5–8, *p < 0.05, **p < 0.01, ***p < 0.001, ****p < 0.0001 vs. FVB by unpaired Welch’s t-test or Mann-Whitney test. Abbreviations: nicotinamide mononucleotide, NMN; nicotinamide riboside, NR; nicotinamide, NAM. Created in https://BioRender.com.

Aortic-surface NMN hydrolysis was significantly increased in Tgαq*44 mice at 14 months of age compared with controls, although an upward trend was also observed at 4 and 12 months (Figures 3I,J). Consistent with previous results, NAD^+^-related metabolites, including NAM and NR, were released in significantly higher amounts by Tgαq*44 aortas at 12 and 14 months following incubation with NMN (Figures 3K,L). Collectively, these data suggest enhanced extracellular NAD^+^ catabolism at the aortic surface of older Tgαq*44 mice. This is associated with increased NAM generation and may also be accompanied by activation of additional ecto-enzymes, resulting in elevated NR production. Notably, increased NAD^+^ catabolism was observed in aortic fragments from Tgαq*44 mice at the advanced stage of HF.

Despite accelerated extracellular NAD^+^ turnover, no disturbances in energy or redox metabolism were observed in aortic segments from 14-month-old Tgαq*44 mice (Supplementary Figure S1, Supplementary Material). Intracellular levels of NAD^+^, total NAD (NAD^+^+NADH; tNAD), NADP^+^, and IMP showed an increasing trend in Tgαq*44 mice compared with controls. No statistically significant differences were observed in ATP or ADP levels. AMP was significantly decreased in Tgαq*44 aorta, but no overall alterations in TAN or AEC were detected. In turn, total guanine nucleotides (TGN) were significantly increased, driven by higher levels of GMP, GDP, and GTP in Tgαq*44 aortas compared to controls.

### 3.3 Aortic CD38 abundance and a low-molecular-weight VCAM-1-immunoreactive band were increased in end-stage HF in Tgαq*44 mice

To determine whether enhanced aortic NAD^+^ and NMN degradation was accompanied by increased CD38 abundance, we quantified CD38 protein in aortas from 14-month-old mice. CD38 abundance was significantly higher in Tgαq*44 aortas than in age-matched FVB controls (Figure 4A). To assess concomitant vascular inflammatory activation, we measured ICAM-1 and VCAM-1 abundance. Two VCAM-1-immunoreactive bands were detected: a high-molecular-weight band (∼115 kDa) and a low-molecular-weight band (∼45 kDa; Figure 4B). The low-molecular-weight band was significantly increased in Tgαq*44 aortas, a pattern consistent with inflammation-associated alternative splicing (Hahne et al., 1994). The high-molecular-weight VCAM-1 band showed a non-significant increasing trend, whereas ICAM-1 abundance did not differ between groups. In contrast, no differences in aortic CD38, VCAM-1, or ICAM-1 protein levels were observed between 4-month-old Tgαq*44 and FVB mice (Supplementary Figure S2, Supplementary Material). This finding is in line with the unchanged aortic NAD^+^ turnover observed in the corresponding enzymatic assay and suggests that early-stage HF is not accompanied by detectable vascular inflammatory activation, as assessed by vascular adhesion molecule abundance.

**FIGURE 4:**
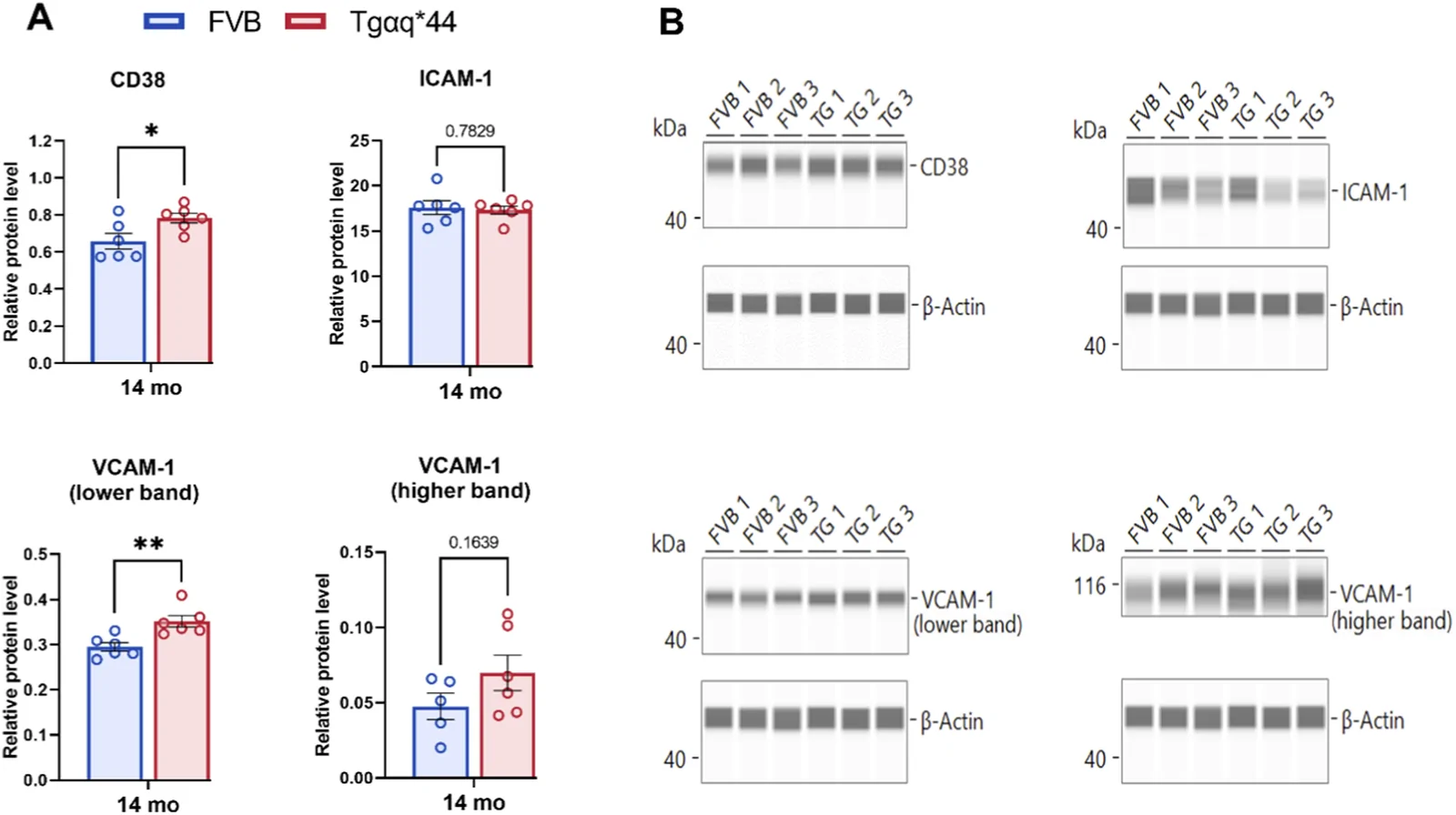
Elevated levels of CD38 and the low-molecular-weight VCAM-1-immunoreactive band in the aorta of 14-month-old Tgαq*44 mice. **(A)** Quantification of relative protein levels normalized to β-actin. **(B)** Representative immunoblots showing CD38, ICAM-1, and the low- and high-molecular-weight VCAM-1-immunoreactive bands, with β-actin used as a loading control. Data are presented as mean ± SEM; n = 5–6, with exact p value or *p < 0.05; **p < 0.01 vs. FVB by unpaired Welch’s t- or Mann-Whitney test.

### 3.4 Perturbed arginine-NO metabolism in Tgαq*44 mice

To characterize the genotype-specific differences in the AA profile across the analyzed age range, we performed targeted serum AA mapping. PCA revealed clear separation of AA profiles between Tgαq*44 and FVB mice at 4, 12, and 14 months, with partial overlap at 8 months (Figure 5A). At 4 months, Tgαq*44 mice exhibited an overall reduction in the AA pool, primarily driven by lower concentrations of lysine, glutamic acid, aspartic acid, BCAAs (isoleucine, leucine, and valine), and methylated arginine derivatives, including asymmetric dimethylarginine (ADMA), symmetric dimethylarginine (SDMA), and N-monomethyl-L-arginine (L-NMMA; Figure 5B). In contrast, these mice tended to display higher serum levels of citrulline, arginine, and tryptophan, a precursor of *de novo* NAD^+^ synthesis, with the latter remaining significantly elevated also at the advanced stage of HF (12–14 months). At 8 months, the separation between genotypes was minimal and was primarily driven by changes in the arginine/NO axis and BCAA metabolism that were opposite to those observed at 4 months, along with a marked increase in tyrosine levels in Tgαq*44 mice compared with FVB controls. In 12–14-month-old mice, the Tgαq*44 AA profile was characterized by widespread alterations affecting most AAs, with prominent contributions from pathways related to arginine, BCAA, aromatic AAs, and nitrogen metabolism. Notably, several metabolites, including ornithine, citrulline, proline, and methionine, displayed divergent patterns between the 12- and 14-month Tgαq*44 cohorts. Overall, alterations in the AA profile of Tgαq*44 mice appeared to be stage-dependent, with the most pronounced metabolic shift observed at 12 months, followed by further reorganization at the late stage of HF (14 months).

**FIGURE 5:**
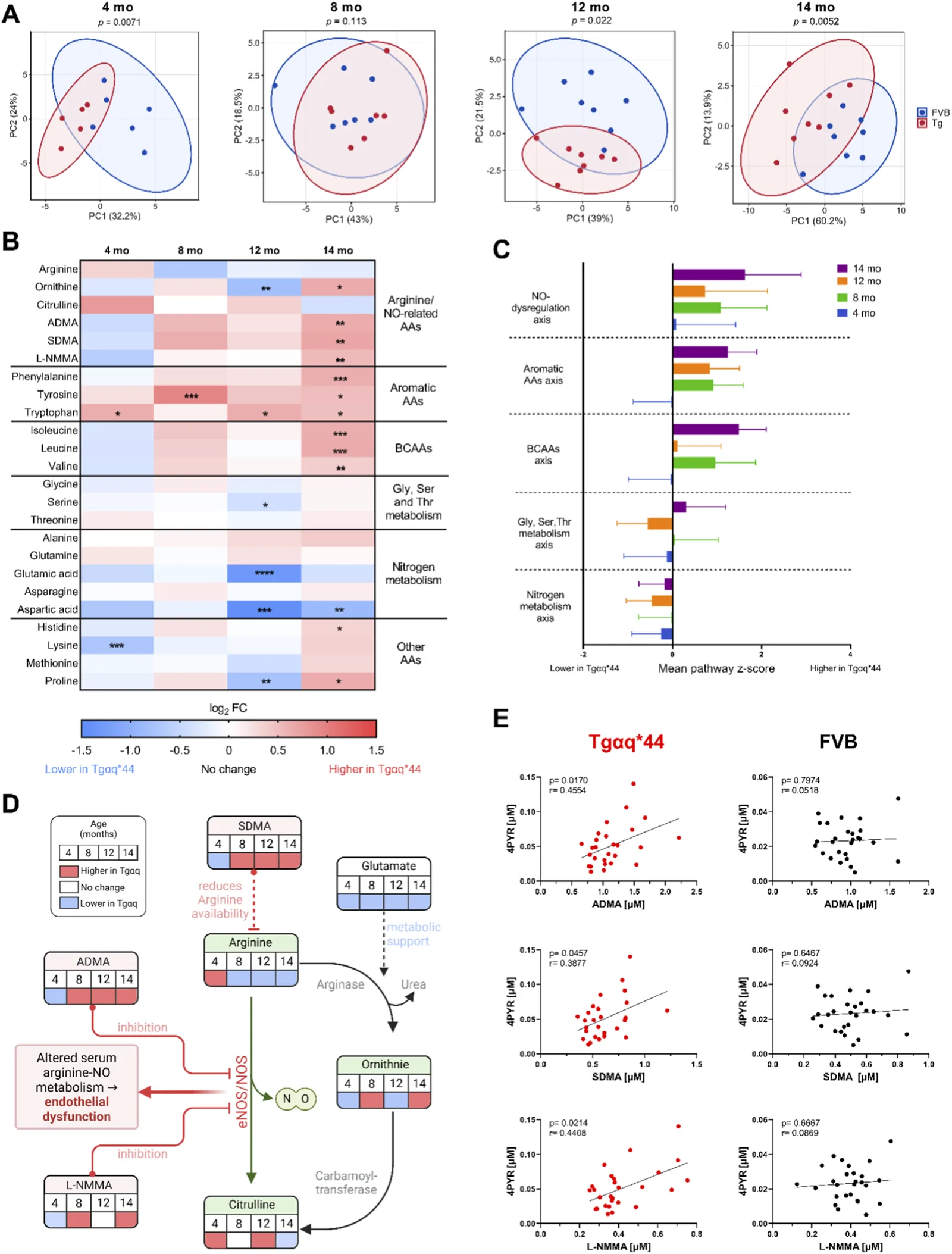
Altered serum amino acid (AA) signatures in Tgαq*44 mice are linked with dysregulated arginine-NO metabolism. **(A)** Principal component analysis (PCA) score plots of serum amino acid profiles in FVB and Tgαq*44 mice at 4, 8, 12 and 14 months of age. **(B)** Heatmap showing log_2_ fold changes (FC) in serum amino acid concentrations across age groups, with metabolites assigned to pathway-related modules. **(C)** Forest plot of module score differences between Tgαq*44 and FVB mice. **(D)** Schematic representation of the arginine/NO-related metabolic axis in Tgαq*44 mice relative to FVB. **(E)** Correlation analysis between methylarginines related to NO metabolism, including ADMA, SDMA, and L-NMMA, and the NAD^+^-related endothelial toxin 4PYR. Bars indicate mean ±95% confidence intervals; n = 5–8 **(A–C)**, n = 20–28 **(E)**; *p < 0.05; **p < 0.01; ***p < 0.001 vs. FVB by unpaired Welch’s t-test or Mann-Whitney test. For PCA, differences between groups were assessed by permutational multivariate analysis of variance (PERMANOVA), with *p*-values indicated above each plot. Red indicates statistically significant correlations determined by Pearson’s or Spearman’s correlation analysis **(E)**. Abbreviations: asymmetric dimethylarginine, ADMA; symmetric dimethylarginine, SDMA; N-monomethyl-L-arginine, L-NMMA; branched-chain amino acids, BCAAs. Created in https://BioRender.com.

To determine which AA-related axes most strongly characterized the Tgαq*44 metabolic phenotype, we performed a pathway-related module analysis. Considering all modules, the NO-dysregulation emerged as one of the strongest cumulative contributors, particularly at the advanced stage of HF (Figure 5C). In addition, aromatic AA and BCAA metabolism displayed high cumulative effect sizes, indicating their contribution to the metabolic phenotype of Tgαq*44 mice. However, the effect size of the NO-dysregulation module was larger in Tgαq*44 mice at each analyzed age compared with controls. Based on the schematic representation, at earlier stages of disease progression, changes within the arginine-citrulline axis were accompanied by relatively low levels of NO synthase (NOS) inhibitors and preserved arginine availability (Figure 5D). However, this profile changed markedly between 12 and 14 months of age in Tgαq*44 mice, with a shift toward lower arginine availability and increased concentrations of methylarginines, including ADMA, SDMA, and L-NMMA. ADMA and L-NMMA are endogenous NOS inhibitors, whereas SDMA may indirectly influence NO metabolism through effects on arginine transport. Collectively, this pattern is consistent with disturbed arginine-NO metabolism.

To further assess the relationship between NO pathway dysregulation and endothelial dysfunction, we correlated serum ADMA, SDMA, and L-NMMA levels with NAD^+^-related endothelial toxin 4PYR (Figure 5E). Positive correlations between all three methylarginines and 4PYR concentration were found only in Tgαq*44 mice. These associations are consistent with coordinated remodeling of 4PYR and arginine/NO-related metabolism in Tgαq*44 mice, but do not establish a causal link between 4PYR build-up and impaired endothelial function.

## 4 Discussion

In this study, we demonstrated that HF progression in Tgαq*44 mice was accompanied by systemic NAD^+^ depletion and a shift toward a vascular NAD^+^-catabolic phenotype. Building on our previous findings of myocardial NAD^+^ deficiency in this model, we found that whole-blood NAD^+^ levels decreased early during cardiac dysfunction and declined further as HF progressed. This systemic NAD^+^ deficit coincided with enhanced extracellular catabolism of NAD^+^ and NMN at the aortic surface, elevated aortic CD38 abundance, perturbations in metabolites related to NO-mediated vascular function, and an increase in a low-molecular-weight VCAM-1-immunoreactive band. These findings suggest that enhanced aortic NAD^+^ catabolism may represent a vascular component of HF progression rather than a passive consequence of myocardial metabolic failure.

Tgαq*44 mice develop slowly progressing chronic HF driven by cardiomyocyte-specific Gαq overexpression and chronic neurohumoral overstimulation (Mende et al., 2001). Given its mixed systolic–diastolic phenotype, the Tgαq*44 model does not directly correspond to clinically defined, EF-based HF subtypes; instead, its disease course is defined by model-specific, age-dependent deterioration of multiple functional and structural cardiac parameters, culminating in a late/end-stage HF phenotype at 12–14 months (Olkowicz et al., 2026). Consistent with previous reports, we observed progressive systolic dysfunction and structural remodeling, confirming the suitability of this model for studying metabolic alterations across the transition from early dysfunction to overt HF (Berkowicz et al., 2023; Tyrankiewicz et al., 2021a). In prior work, we showed that this progression is accompanied by marked depletion of the myocardial NAD^+^ pool (Olkowicz et al., 2026). The present findings extend this observation beyond the heart by demonstrating an early decrease in whole-blood NAD^+^ that becomes more pronounced at the advanced stage and correlates with impaired systolic function. Thus, NAD^+^ depletion in Tgαq*44 mice appears not to be restricted to the myocardium but to form part of a broader systemic metabolic phenotype. However, because whole blood largely reflects the nucleotide status of circulating cells, these data should be interpreted as evidence of systemic NAD^+^ imbalance. This is particularly relevant to erythrocytes, which predominantly depend on NAD^+^ for glycolytic ATP production, and to leukocytes, in which reduced NAD^+^ availability may affect mitochondrial respiration and immune response, including neutrophil respiratory burst and cytokine production by monocytes and lymphocytes (Hikosaka et al., 2014; Roberts et al., 2013). Conversely, activated immune cells may accelerate NAD^+^ turnover through intracellular PARP-dependent consumption and CD38-mediated hydrolysis of extracellular NAD^+^ (Cameron et al., 2019; Chini et al., 2020).

To further explore whether NAD^+^ depletion was accompanied by altered NAD^+^ availability, we analyzed circulating NAD^+^ precursors and NAM-derived metabolites. At the advanced stage of HF, Tgαq*44 mice showed reduced levels of NA and a tendency toward lower NAM concentrations, consistent with lower circulating availability of substrates for the Preiss-Handler and salvage pathways. Evidence from *in vivo* and clinical studies has confirmed that the NAD^+^ salvage pathway is profoundly altered in HF, resulting in NAD^+^ deficiency and limiting its cellular functions (Tong et al., 2021; Doan et al., 2024). This may be driven by downregulation of NAMPT, as observed in our previous study (Olkowicz et al., 2026), or by increased methylation of NAM associated with impaired autophagic flux (Zhang et al., 2024). In contrast, serum tryptophan was increased, indicating that systemic NAD^+^ depletion was unlikely to result from a generalized shortage of all NAD^+^ precursors. Notably, we observed progressive accumulation of 4PYR, whereas MNA and methylated pyridone metabolites did not show a parallel increase. This is particularly relevant in the vascular context, since 4PYR has been described as an endothelial-toxic derivative capable of impairing cellular energetics and reducing arginine availability for NO production (Pelikant-Malecka et al., 2017; Mierzejewska et al., 2021; Pelikant-Małecka et al., 2014). In Tgαq*44 mice, progressive 4PYR accumulation occurred in parallel with dysregulation of the arginine-NO axis. The observed correlations between 4PYR and methylarginine concentrations further support an association between systemic NAD^+^ imbalance and endothelial dysfunction. Collectively, this pattern suggests that HF progression is associated not only with reduced NAD^+^ availability but also with remodeling of NAM catabolism toward the accrual of potentially harmful products. These findings also provide a rationale for further investigating NAD^+^ precursor supplementation, including NR and NMN. Previous preclinical studies in models of ischemic injury or genetic cardiomyopathy have shown that NR and NMN supplementation can replenish NAD^+^ levels and improve cardiac function and mitochondrial integrity (Tannous et al., 2024; Zhao et al., 2025). However, their effects in Tgαq*44 mice remain unknown and require direct evaluation from both cardiac and vascular perspectives.

A plausible mechanism contributing to systemic and vascular NAD^+^ imbalance is enhanced extracellular degradation of NAD^+^. In the present study, aortic fragments from Tgαq*44 mice with advanced HF displayed increased degradation of both NAD^+^ and NMN. This was accompanied by enhanced release of NAM and NR, whereas NMN, AMP, and adenosine generation did not increase in parallel. The preferential generation of NAM and NR, without increases in NMN, AMP, or adenosine, suggests remodeling toward NAD^+^- and NMN-catabolic pathways producing NAM-related products. Together with increased aortic CD38 abundance, this pattern is consistent with a contribution of CD38-associated extracellular NAD^+^ catabolism in 14-month-old Tgαq*44 mice. Because CD38 is a major ecto-NADase, highly expressed in endothelial cells, and has been linked to NAD^+^/NADP(H) depletion, oxidative stress, and impaired endothelial function (Qiu et al., 2023; Boslett et al., 2018; Agorrody et al., 2022; Zhang et al., 2020; Reyes et al., 2015), our findings support the involvement of CD38-associated extracellular NAD^+^ catabolism in the vascular remodeling observed in late-stage of HF. By contrast, at the early disease stage, the accumulation of NAM and NA alongside a reduced blood NAD^+^ concentration was not accompanied by accelerated aortic NAD^+^ turnover or increased aortic CD38 abundance. This early metabolic imbalance, which preceded advanced HF, may instead reflect inefficient precursor utilization through the salvage and Preiss-Handler pathways, altered interorgan precursor trafficking, and/or increased NAD^+^ consumption by other enzymes or in tissues other than the aorta (Zhang et al., 2019; Chellappa et al., 2022), possibilities that warrant further investigation.

The vascular relevance of this NAD^+^-catabolic phenotype is further suggested by concomitant disturbances in arginine-NO metabolism. Decreased endothelial NO production has been well documented in the Tgαq*44 model (Mohaissen et al., 2022) and is consistent with other observations in HF (Falls et al., 2025; Giannitsi et al., 2019). In the present study, Tgαq*44 mice showed increased circulating concentrations of ADMA, SDMA, and L-NMMA, together with alterations in citrulline and ornithine levels. ADMA and L-NMMA inhibit NOS and are metabolized predominantly through dimethylarginine dimethylaminohydrolase (DDAH)-dependent pathways, whereas SDMA follows distinct metabolic and clearance pathways and may indirectly affect NO production by limiting cellular arginine transport. Thus, the observed profile is indicative of methylarginine imbalance. Reduced expression or activity of DDAH1 may result from genetic factors (Valkonen et al., 2005), metabolic and inflammatory stressors (Ito et al., 1999; Sinisalo et al., 1999), or hemodynamic stress in the heart (Chen et al., 2003). In turn, reduced NOS activity compromises cardiac stress adaptation and signaling, impairs coronary vasodilatory function, and promotes reactive oxygen species generation (Liu et al., 2016). Clinically, increased ADMA levels have been associated with higher mortality and adverse cardiovascular events, establishing ADMA as an independent predictor of new-onset HF (Wirth et al., 2017; Pan et al., 2020). Moreover, L-NMMA may contribute to cytokine-mediated upregulation of VCAM-1 and ICAM-1 expression in endothelial cells, thereby promoting leukocyte adhesion and transmigration (Khan et al., 1996). Specifically, ICAM-1 promotes neutrophil recruitment, whereas VCAM-1 is central to monocyte and lymphocyte activation (Yang et al., 2005; Zohlnhöfer et al., 2000). In our study, however, only the low-molecular-weight VCAM-1-immunoreactive band was increased in aortas from 14-month-old Tgαq*44 mice, whereas ICAM-1 remained unchanged. This selective VCAM-1 response suggests a vascular inflammatory activation compatible with endothelial dysfunction rather than a broad acute inflammatory response in HF (McDowell et al., 2025; Patel et al., 2020). Consistent with this interpretation, a low-molecular-weight VCAM-1-immunoreactive band has been reported to be induced in endothelial cells in response to strong inflammatory stimuli, including tumour necrosis factor-α, interleukin-1β or lipopolysaccharide (Hahne et al., 1994; Garton et al., 2003; Terry et al., 1993). Such a proinflammatory state may be driven by left ventricular dysfunction, through hypertrophy and fibrosis, or by endothelial dysfunction, through apoptosis and reduced NO synthesis, both of which contribute to myocardial failure (Mann, 2002). Taken together, increased 4PYR, enhanced aortic NAD^+^ turnover, impaired arginine/NO metabolism, and selective VCAM-1 upregulation are consistent with vascular metabolism and inflammatory remodeling associated with endothelial dysfunction in advanced HF in Tgαq*44. Nevertheless, further investigation is required to define the specific molecular drivers of this process.

Altered blood adenine nucleotide balance may contribute to this vascular phenotype. We previously reported persistent deficiency of high-energy phosphates in Tgαq*44 hearts during both early and late stages of HF (Olkowicz et al., 2026). Here, reduced whole-blood ATP levels and ATP/ADP ratio, together with increased AMP, indicate altered adenine nucleotide balance in circulating cells, particularly erythrocytes, rather than a direct measure of myocardial bioenergetics. This may be relevant to endothelial function, since circulating ATP stimulates NO production and adenosine generation, thereby contributing to vasodilatory signaling (Sprague et al., 1996). Moreover, ageing Tgαq*44 mice were previously shown to develop erythropathy, suggesting a bidirectional relationship between altered red blood cell function and endothelial dysfunction (Mohaissen et al., 2022). Therefore, the whole-blood adenine nucleotide profile should be interpreted as an additional readout of circulating cells that may support impaired vascular function in HF. The accompanying changes in circulating BCAAs are consistent with the broader metabolic remodeling previously described in Tgαq*44 mice and patients with HF (Olkowicz et al., 2026; Tobias et al., 2018; Ruiz-Canela et al., 2016; Sun et al., 2016; Wang et al., 2016; Li et al., 2017). Although BCAAs may be associated with the activation of endothelial NOS (D’Antona et al., 2010), in the present study, they are best viewed as a parallel marker of disease progression rather than as a primary component of the vascular NAD^+^ phenotype.

Finally, the distinct nucleotide pattern of aortic tissue suggests additional vascular purine remodeling in advanced HF. Although we did not detect a significant imbalance in the aortic NAD^+^/NADH ratio, Tgαq*44 mice showed an increased guanine nucleotide pool, reflected by higher GMP, GDP, and GTP levels. Because endothelial NO production and protective responses depend on intracellular redox, energetic, and purinergic balance (Wilson et al., 2019; Coutelle et al., 2014; Lapel et al., 2017; Xiao et al., 2021), these changes may indicate a compensatory response to meet metabolic demands in the vascular wall. Previous metabolomic studies have linked endothelial activation to changes in AMP and guanine metabolism (Jayaraman et al., 2018). Moreover, GTP-dependent signaling participates in endothelial permeability, angiogenic responses, and G-protein-mediated pathways (Lutz et al., 2001; Feng et al., 2014; Huang et al., 2022). Thus, increased aortic guanine nucleotides may provide an additional indication of vascular changes in Tgαq*44 mice, although this finding requires functional validation.

Several limitations of the present study should be considered. First, although increased aortic NAD^+^/NMN degradation accompanied by elevated CD38 abundance supports a role for CD38 in vascular NAD^+^ catabolism, a causal relationship was not directly established, as neither pharmacological inhibition nor genetic deletion of CD38 was examined. Second, because aortic tissue samples comprise multiple vascular cell types, the observed alterations should be interpreted as changes within the vascular wall, potentially involving endothelial cells, rather than as endothelial cell-specific effects. Furthermore, because perivascular adventitial tissue was removed before *ex vivo* incubation, this experimental model does not fully reflect the intact vascular microenvironment. Third, NO production, endothelial NOS activity, and DDAH activity were not assessed directly. Consequently, conclusions regarding impaired NO signaling rely on indirect evidence derived from circulating arginine-NO pathway metabolites and markers of endothelial activation. Fourth, whole-blood nucleotide measurements primarily reflect the adenylate and NAD^+^ status of circulating blood cells, particularly erythrocytes and leukocytes, and therefore should not be considered a direct indicator of myocardial bioenergetics, as previously discussed (Olkowicz et al., 2026). Finally, the study was conducted exclusively in female Tgαq*44 mice; therefore, potential sex-specific differences were not assessed, which may limit the generalizability of the findings across sexes. Sex-related differences in HF extend beyond disease prevalence and include distinct phenotypic presentation and clinical trajectories (Valente et al., 2026). Women are disproportionately represented among patients with HF with preserved ejection fraction (HFpEF), whereas HFrEF is more common in men. Although women with HF generally show more favourable survival than men, they may experience greater functional limitation and poorer quality of life. Consequently, the exclusive use of female Tgαq*44 mice should be considered when extrapolating the present findings across sexes, particularly because sex-dependent differences may influence vascular dysfunction, metabolic remodelling, and the relative contribution of systolic and diastolic abnormalities.

In conclusion, Tgαq*44 mice exhibited progressive remodeling of systemic and vascular NAD^+^ metabolism during HF development. Beyond the previously reported myocardial NAD^+^ depletion, this phenotype was characterized by reduced circulating NAD^+^ availability, altered profiles of NAD^+^ precursors and nicotinamide-derived metabolites, enhanced aortic NAD^+^/NMN degradation, increased aortic CD38 abundance accompanied by selective VCAM-1 upregulation, and disturbances in arginine-NO metabolism. Collectively, these findings identified vascular NAD^+^ catabolism as a previously underappreciated component of HF progression in Tgαq*44 mice. The association between increased aortic CD38 abundance and enhanced NAD^+^ degradation further implicates CD38 as a potential mediator of this process and a candidate target for future mechanistic and interventional studies.

## Data Availability

The raw data supporting the conclusions of this article will be made available by the authors, without undue reservation.
