# Supplementary material for: Circulating and vascular NAD+ metabolism remodeling in Tgαq*44 mice with progressive heart failure
Source: Front Mol Biosci. 2026 Oct 9;13:1927197. doi: 10.3389/fmolb.2026.1927197 (PMC13690639; doi:10.3389/fmolb.2026.1927197)
Supplement: Supplementary file 1 [file DataSheet1.docx]

Supplementary Material

**Circulating and Vascular NAD⁺ Metabolism Remodeling in Tgαq*44 Mice With Progressive Heart Failure**

**Agata Jedrzejewska^1^, Oliwia Krol^1^,, Patryk Mucha^1^, Ada Kawecka^1^, Ewa Slominska^1^, Mariola Olkowicz^2,3^, Ryszard T. Smolenski^1^, Marta Tomczyk^1*^**

^1^ Department of Biochemistry, Medical University of Gdansk, Debinki 1, 80-211 Gdansk, Poland

^2^ Department of Biopharmaceutics and Pharmacodynamics, Medical University of Gdansk, Hallera 107, 80-416 Gdansk, Poland

^3^ Jagiellonian Centre for Experimental Therapeutics (JCET), Jagiellonian University, Bobrzynskiego 14, 30-348 Krakow, Poland

*** Correspondence:**
Marta Tomczyk
[marta.tomczyk@gumed.edu.pl](mailto:marta.tomczyk@gumed.edu.pl)

**Supplementary Table 1.** Left ventricular (LV) mass and body weight of Tgαq*44 and FVB mice at different months (mo). Data are presented as mean ± SEM; *p < 0.05, **p < 0.01 vs. FVB by unpaired Student’s t-test with Welch’s correction or Mann–Whitney test.

|  | **FVB** | | | | **Tgαq*44** | | | |
| --- | --- | --- | --- | --- | --- | --- | --- | --- |
|  | 4 mo  *n=6* | 8 mo  *n=7* | 12 mo  *n=8* | 14 mo  *n=8* | 4 mo  *n=5* | 8 mo  *n=7* | 12 mo  *n=7* | 14 mo  *n=8* |
| **BW**  **(g)** | 26.12 ± 0.94 | 28.69 ± 1.34 | 27.08 ± 0.98 | 31.49 ± 0.98 | 24.22 ± 0.70 | 28.96 ± 1.03 | 28.89 ± 1.39 | 29.03 ± 0.33  * |
| **LV mass**  **(mg)** | 105.6 ± 4.75 | 138.5 ± 5.64 | 142.5 ± 4.79 | 139.5 ± 5.21 | 123.3 ± 5.77  * | 144.6 ± 5.73 | 158.8 ± 14.07 | 195.7 ± 14.94  ** |

| **Parameters** | **FVB** | | | | | | | | | | | | | | | | **Tgαq*44** | | | | | | | | | | | | | | | | |
| --- | --- | --- | --- | --- | --- | --- | --- | --- | --- | --- | --- | --- | --- | --- | --- | --- | --- | --- | --- | --- | --- | --- | --- | --- | --- | --- | --- | --- | --- | --- | --- | --- | --- |
|  | **NAD^+^** | | **NADH** | | **NAD^+^/ NADH** | | **NADP^+^** | | **ATP** | | **ADP** | | **ATP/ ADP** | | **AMP** | | **NAD^+^** | | **NADH** | | **NAD^+^/ NADH** | | **NADP^+^** | | **ATP** | | **ADP** | | **ATP/ ADP** | | **AMP** | |  |
|  | ***r*** | ***p*** | ***r*** | ***p*** | ***r*** | ***p*** | ***r*** | ***p*** | ***r*** | ***p*** | ***r*** | ***p*** | ***r*** | ***p*** | ***r*** | ***p*** | ***r*** | ***p*** | ***r*** | ***p*** | ***r*** | ***p*** | ***r*** | ***p*** | ***r*** | ***p*** | ***r*** | ***p*** | ***r*** | ***p*** | ***r*** | ***p*** |  |
| **IVSd**  **(mm)** | 0.29 | ns | 0.17 | ns | -0.24 | ns | 0.31 | ns | -0.13 | ns | 0.26 | ns | -0.23 | ns | 0.16 | ns | -0.04 | ns | -0.33 | ns | 0.24 | ns | -0.05 | ns | 0.05 | ns | -0.15 | ns | 0.16 | ns | -0.24 | ns |  |
| **LVIDd (mm)** | -0.27 | ns | 0.16 | ns | -0.18 | ns | -0.10 | ns | -0.31 | ns | 0.19 | ns | -0.24 | ns | 0.21 | ns | -0.12 | ns | 0.18 | ns | -0.24 | ns | -0.01 | ns | -0.19 | ns | -0.22 | ns | -0.05 | ns | 0.15 | ns |  |
| **LVPWd (mm)** | 0.06 | ns | -0.02 | ns | -0.05 | ns | -0.07 | ns | -0.15 | ns | 0.04 | ns | -0.21 | ns | 0.08 | ns | -0.24 | ns | -0.10 | ns | 0.06 | ns | 0.14 | ns | -0.26 | ns | -0.16 | ns | -0.10 | ns | 0.06 | ns |  |
| **IVSs (mm)** | 0.21 | ns | -0.09 | ns | 0.02 | ns | 0.27 | ns | 0.15 | ns | 0.14 | ns | 0.01 | ns | -0.08 | ns | -0.11 | ns | -0.29 | ns | 0.24 | ns | 0.02 | ns | 0.03 | ns | -0.05 | ns | 0.11 | ns | -0.16 | ns |  |
| **LVIDs (mm)** | -0.13 | ns | 0.19 | ns | -0.20 | ns | 0.01 | ns | -0.31 | ns | 0.18 | ns | -0.25 | ns | 0.22 | ns | -0.23 | ns | 0.33 | ns | -0.46 | * | -0.26 | ns | -0.44 | * | -0.18 | ns | -0.28 | ns | 0.28 | ns |  |
| **LVPWs (mm)** | 0.23 | ns | 0.04 | ns | -0.10 | ns | 0.02 | ns | 0.12 | ns | 0.27 | ns | -0.29 | ns | 0.15 | ns | 0.14 | ns | -0.49 | * | 0.44 | ns | 0.36 | ns | 0.16 | ns | -0.12 | ns | 0.21 | ns | -0.34 | ns |  |
| **EF (%)** | -0.16 | ns | -0.33 | ns | 0.16 | ns | -0.23 | ns | 0.29 | ns | -0.07 | ns | 0.19 | ns | -0.32 | ns | 0.54 | * | -0.60 | ** | 0.65 | ** | 0.54 | * | 0.68 | *** | 0.04 | ns | 0.55 | * | -0.59 | ** |  |
| **FS (%)** | -0.16 | ns | -0.33 | ns | 0.19 | ns | -0.23 | ns | 0.27 | ns | -0.09 | ns | 0.20 | ns | -0.32 | ns | 0.54 | * | -0.60 | ** | 0.63 | ** | 0.53 | * | 0.67 | ** | 0.04 | ns | 0.54 | * | -0.59 | ** |  |
| **RWT** | 0.09 | ns | -0.01 | ns | 0.01 | ns | -0.01 | ns | -0.10 | ns | -0.02 | ns | -0.10 | ns | 0.06 | ns | -0.17 | ns | -0.10 | ns | 0.13 | ns | 0.11 | ns | -0.17 | ns | -0.01 | ns | -0.11 | ns | 0.06 | ns |  |
| **LVMI** | 0.03 | ns | 0.03 | ns | -0.08 | ns | 0.12 | ns | 0.01 | ns | 0.25 | ns | -0.22 | ns | 0.17 | ns | 0.11 | ns | -0.23 | ns | 0.06 | ns | 0.20 | ns | -0.08 | ns | -0.16 | ns | 0.03 | ns | -0.02 | ns |  |

**Supplementary Table 2. Correlations between echocardiographic parameters and circulating nucleotide levels in FVB and Tgαq*44 blood.** Data are presented as correlation coefficients (r) with corresponding p-values (p); ns indicates non-significant; n = 19-27, *p < 0.05, **p < 0.01, and ***p < 0.001.

**Supplementary Table 3. Correlations between echocardiographic parameters and serum levels of NAD^+^ precursors and its metabolites in FVB and Tgαq*44 blood.** Data are presented as correlation coefficients (r) with corresponding p-values (p); ns indicates non-significant; n = 19-27, *p < 0.05, **p < 0.01.

| **Parameters** | **FVB** | | | | | | | | | | | | **Tgαq*44** | | | | | | | | | | | | |
| --- | --- | --- | --- | --- | --- | --- | --- | --- | --- | --- | --- | --- | --- | --- | --- | --- | --- | --- | --- | --- | --- | --- | --- | --- | --- |
|  | **NAM** | | **NA** | | **MNA** | | **4PYR** | | **Met2PY** | | **Met4PY** | | **NAM** | | **NA** | | **MNA** | | **4PYR** | | **Met2PY** | | **Met4PY** | |  |
|  | ***r*** | ***p*** | ***r*** | ***p*** | ***r*** | ***p*** | ***r*** | ***p*** | ***r*** | ***p*** | ***r*** | ***p*** | ***r*** | ***p*** | ***r*** | ***p*** | ***r*** | ***p*** | ***r*** | ***p*** | ***r*** | ***p*** | ***r*** | ***p*** |  |
| **IVSd**  **(mm)** | 0.09 | ns | 0.43 | * | 0.19 | ns | 0.24 | ns | 0.20 | ns | 0.20 | ns | -0.08 | ns | 0.27 | ns | -0.31 | ns | 0.16 | ns | -0.18 | ns | -0.17 | ns |  |
| **LVIDd (mm)** | 0.04 | ns | 0.05 | ns | -0.18 | ns | 0.23 | ns | -0.24 | ns | -0.20 | ns | -0.31 | ns | 0.16 | ns | 0.34 | ns | 0.25 | ns | 0.32 | ns | 0.35 | ns |  |
| **LVPWd (mm)** | 0.13 | ns | -0.01 | ns | -0.13 | ns | 0.03 | ns | 0.01 | ns | 0.002 | ns | -0.33 | ns | 0.20 | ns | 0.13 | ns | 0.18 | ns | 0.07 | ns | 0.13 | ns |  |
| **IVSs (mm)** | 0.26 | ns | 0.25 | ns | 0.11 | ns | 0.18 | ns | 0.07 | ns | 0.10 | ns | -0.18 | ns | 0.39 | ns | -0.33 | ns | 0.05 | ns | -0.10 | ns | -0.06 | ns |  |
| **LVIDs (mm)** | 0.02 | ns | 0.19 | ns | -0.17 | ns | 0.25 | ns | -0.16 | ns | -0.13 | ns | -0.51 | * | 0.10 | ns | 0.29 | ns | 0.39 | ns | 0.22 | ns | 0.26 | ns |  |
| **LVPWs (mm)** | 0.05 | ns | -0.25 | ns | 0.19 | ns | -0.20 | ns | 0.15 | ns | 0.14 | ns | 0.27 | ns | -0.14 | ns | 0.12 | ns | -0.29 | ns | 0.08 | ns | 0.08 | ns |  |
| **EF (%)** | 0.03 | ns | -0.29 | ns | 0.08 | ns | -0.22 | ns | -0.10 | ns | -0.09 | ns | 0.67 | ** | -0.25 | ns | -0.08 | ns | -0.67 | ** | 0.02 | ns | -0.02 | ns |  |
| **FS (%)** | 0.04 | ns | -0.29 | ns | 0.08 | ns | -0.23 | ns | -0.10 | ns | -0.09 | ns | 0.65 | ** | -0.25 | ns | -0.10 | ns | -0.67 | ** | 0.01 | ns | -0.03 | ns |  |
| **RWT** | 0.09 | ns | -0.05 | ns | -0.01 | ns | -0.07 | ns | 0.12 | ns | 0.10 | ns | -0.18 | ns | 0.16 | ns | -0.04 | ns | 0.13 | ns | -0.06 | ns | -0.02 | ns |  |
| **LVMI** | 0.11 | ns | 0.11 | ns | -0.17 | ns | 0.02 | ns | -0.13 | ns | -0.07 | ns | -0.48 | * | 0.43 | ns | -0.09 | ns | 0.13 | ns | 0.02 | ns | 0.08 | ns |  |

**Supplementary Figure 1. Increased level of guanine nucleotide pool in the aorta of the Tgαq*44 mice in the advanced heart failure stage.** Total and components of NAD^+^ and NADP^+^ pools (**A**), total and components of adenine nucleotide pool (**B**), total and components of guanine nucleotide pool (**C**), IMP concentration (**D**) in aortas of FVB and Tgαq*44 mice. Data are presented as mean ± SEM; n = 6-8, *p < 0.05 vs. FVB by unpaired Student’s t-test with Welch’s correction. Abbreviations: T NAD, total NAD^+^ pool (NAD+NADH); TAN, total adenine nucleotide pool (ATP+ADP+AMP); AEC, adenylate energy charge; TGN, total guanine nucleotide pool (GTP+GDP+GMP).

**Supplementary Figure 2. Aortic protein abundance of CD38, intercellular adhesion molecule 1 (ICAM-1), and vascular cell adhesion molecule 1 (VCAM-1) in 4-month-old FVB and Tgαq*44 mice. (A)** Relative protein abundance normalized to glyceraldehyde-3-phosphate dehydrogenase (GAPDH). **(B)** Representative immunoblots showing CD38, ICAM-1, and the lower- and higher-molecular-weight VCAM-1-immunoreactive bands, with GAPDH used as a loading control. Data are presented as mean ± SEM; n = 6 per group, with exact p value performed using unpaired Student’s t-test with Welch’s correction.
